# Supplementary material for: Evaluation of the implementation and effectiveness of a multifactorial intervention strategy for safe patient handling and movement in the healthcare sector: a study protocol of a cluster randomised controlled trial
Source: BMJ Open. 2023 Feb 3;13(2):e067693. doi: 10.1136/bmjopen-2022-067693 (PMC9900050; doi:10.1136/bmjopen-2022-067693)
Supplement: Author's manuscript [file bmjopen-2022-067693.draft_revisions.pdf]

BMJ Open

BMJ Open is committed to open peer review. As part of this commitment we make the peer review history of every article we publish publicly available.

When an article is published we post the peer reviewers' comments and the authors' responses online. We also post the versions of the paper that were used during peer review. These are the versions that the peer review comments apply to.

The versions of the paper that follow are the versions that were submitted during the peer review process. They are not the versions of record or the final published versions. They should not be cited or distributed as the published version of this manuscript.

BMJ Open is an open access journal and the full, final, typeset and author-corrected version of record of the manuscript is available on our site with no access controls, subscription charges or pay-per-view fees (http://bmjopen.bmj.com).

If you have any questions on BMJ Open's open peer review process please email info.bmjopen@bmj.com

BMJ Open

Evaluation of Implementation and Effectiveness of a Multifactorial Intervention Strategy for Safe Patient Handling and Movement in the Healthcare Sector: Study Protocol of a Cluster Randomized Controlled Trial

Journal:	*BMJ Open*
Manuscript ID	bmjopen-2022-067693
Article Type:	Protocol
Date Submitted by the Author:	01-Sep-2022

Complete List of Authors:	Wahlin, Charlotte; Linköping University, Department of Health, Medicine and Caring Sciences; Karolinska Institutet<br>Buck, Sebastian; Linköping University, Department of Health, Medicine and Caring Sciences<br>Sandqvist, Jan; Linköping University, Department of Health, Medicine and Caring Sciences Rehabilitation and Community Medicine<br>Enthoven, Paul; Linkopings universitet, Department of Health, Medicine and Caring Sciences<br>Fock, Jenni; Linköping University Hospital<br>Andreassen, Maria; Linköping University, Department of Health, Medicine and Caring Sciences Rehabilitation and Community Medicine<br>Strid, Emma; Örebro Universitet

Keywords:	Health & safety < HEALTH SERVICES ADMINISTRATION & MANAGEMENT, PUBLIC HEALTH, Musculoskeletal disorders < ORTHOPAEDIC & TRAUMA SURGERY

SCHOLARONE™
Manuscripts

Evaluation of Implementation and Effectiveness of a Multifactorial Intervention Strategy for Safe Patient Handling and Movement in the Healthcare Sector: Study Protocol of a Cluster Randomized Controlled Trial

Charlotte Wåhlin[1, 2], Sebastian Buck[1], Jan Sandqvist[3], Paul Enthoven[4], Jenni Fock[5], Maria Andreassen[3], Emma Nilsing Strid[6]

[1]Occupational and Environmental Medicine Center, Department of Health, Medicine and Caring Sciences, Division of Prevention, Rehabilitation and Community Medicine, Unit of Clinical Medicine, Linköping University, Linköping, Sweden.

[2]Unit of Intervention and Implementation Research for Worker Health, Karolinska Institutet, Stockholm, Sweden.

[3] Department of Health, Medicine and Caring Sciences, Division of Prevention, Rehabilitation and Community Medicine, Unit of Occupational Therapy, Linköping University, Norrköping, Sweden.

[4] Department of Health, Medicine and Caring Sciences, Division of Prevention, Rehabilitation and Community Medicine, Unit of Physiotherapy, Linköping University, Linköping, Sweden.

[5]Linköping University hospital, Region Östergötland, Linköping, Sweden.

[6]University Health Care Research Center, Faculty of Medicine and Health, Örebro University, Örebro, Sweden.

Correspondence to Charlotte Wåhlin: Occupational and Environmental Medicine Center, Linköping University, 58183 Linköping, Sweden.

E-mail: charlotte.wahlin@liu.se

Trial registration ClinicalTrials.gov identifier: NCT05276180

For peer review only

Abstract

Introduction

Healthcare workers (HCWs) with physically demanding work tasks, such as patient handling and movement (PHM), are at high risk of musculoskeletal disorders (MSD). To facilitate safe PHM and prevent MSD, a combination of workplace interventions, including risk assessments, are needed. The aim of this study is to implement and evaluate a multifactorial intervention strategy for safe PHM and compare it with a single intervention strategy.

Methods and analysis

This is a cluster randomized controlled trial (RCT) which will compare a multifactorial intervention strategy with a single intervention strategy for safe PHM in workplaces in the Swedish regional and municipal healthcare systems. At least twelve healthcare units will be recruited. Units belonging to Arm A will receive: 1) guidelines for PHM; 2) training modules; 3) risk assessment with TilThermometer; 4) risk assessment with Downtown Fall Risk Index; and 5) work environment mapping. Units belonging to Arm B will only receive interventions 1) and 5). The two strategies will be evaluated with regard to 1) the primary outcome of the intervention effectiveness of the applied strategies (safety climate in relation to aspects of PHM) and, 2) the primary implementation outcome (acceptability, appropriateness and feasibility). This study will also explore the implementation process and fidelity to the intervention guidelines, examine the influence of contextual factors, and explore participants' experiences of working with strategies for safe PHM. A mix of quantitative and qualitative methods will be used. The data collection is based on questionnaires, interviews and field notes of contextual factors.

For peer review only

Ethics and dissemination

The study is approved by the Swedish national ethical board (Dnr 2021–00578). Study results will be published in peer-reviewed journals, presented at conferences and distributed on social media. A lay summary and dissemination strategy will be codesigned with a reference group and participating healthcare units.

Trial registration Number

NCT05276180

Keywords: Occupational health, implementation science, risk assessment, moving and lifting patients, health care sector, health care workers, musculoskeletal disorders, occupational safety and health, work environment

Strengths and limitations of this study

- Main strengths of this project are the randomized controlled trial design with assessment of both intervention effectiveness and implementation outcomes, and the use of quantitative as well as qualitative methods.
- Findings from this randomized controlled trial will contribute knowledge about the effectiveness and experience of using two different strategies for promoting safe patient handling and movement at workplaces in healthcare settings.
- Validated outcome measures are used to evaluate intervention effectiveness and implementation outcomes.
- The trial findings are limited to workplaces in regional and municipal healthcare and do not include homecare.

For peer review only - http://bmjopen.bmj.com/site/about/guidelines.xhtml

INTRODUCTION

There are several risk factors in the healthcare environment which lead to work-related injuries among healthcare workers (HCWs) and harmful incidents and injuries among patients. In previous research, based on the Swedish Patient and Workers Safety Study (PAWSS), patient injuries and HCW incidents and injuries reported over four years in the healthcare sector were analyzed. The results indicate that both patient safety and occupational safety for HCWs are insufficient (1). Common occupational injuries among healthcare professionals are those caused by sharp objects or needles; exposure to infection; injuries that occur in threatening and violent situations; falls; and injuries that occur during patient handling and movement (PHM) (1-3). Musculoskeletal disorders (MSD) that occur in connection with PHM are one of the most common injuries. A recent study by Keyaerts et al. (4) shows that physical and psychosocial work-related factors, and individual factors like fear of movement, are related to MSDs in HCWs. MSDs are most frequent in the lumbar spine, neck and shoulders among nursing staff, especially nurses and assistant nurses (4-8). The majority of the incidents and injuries among HCWs occur when caring for patients. This demonstrates the importance of combining risk assessments for patients and HCWs to prevent and eliminate risks and to promote a healthy, sustainable healthcare environment. Patient falls are also a cause of injury, with 1860 cases reported (17% of all incident reports) (1). Many patient falls can be prevented by HCWs if they can be taught risk assessment skills to identify factors associated with risk to patients and to themselves and skills to improve safe patient handling and movement (9). Previous research into the relationships between HCWs working conditions and patient injuries demonstrates that a good safety climate supports safe patient care and ensures the safety of the HCWs (10). An interview study of HCWs was carried out within the framework of PAWSS. It analyzed situations in which both patients and HCWs risked injury or were actually injured in the healthcare setting (11). The study shows that organizational support is important for both risk management and coping with the consequences of injuries. There is a need to study risk

For peer review only - http://bmjopen.bmj.com/site/about/guidelines.xhtml

assessment and intervention strategies in the healthcare sector that can facilitate safe PHM. The present study will use the risk assessment instrument TilThermometer to assess HCWs' physical exposure in different types of situations involving PHM. The TilThermometer has been translated from Dutch into Swedish. Its linguistic validity and cross-cultural adaptation have been evaluated and it is available online (12, 13). However, the use of the Swedish version of TilThermometer has not been evaluated in a healthcare setting. Furthermore, a fall risk assessment will be performed using Downton Fall Risk Index (14) or other existing fall risk assessment instruments used in care units to identify whether a patient has a risk of falling.

Within the PAWSS project, a recent systematic review by Wåhlin and co-authors shows that a number of work interventions can be effective for safe PHM (15). These interventions include not only providing work equipment but also training workers, since this can increase their use of equipment. Training workers to be peer coaches is also associated with fewer injuries. Other effective strategies for safe PHM are participatory ergonomics and management engagement with HCWs. The starting point for a safe environment and a strengthened safety climate is that the employer, together with HCWs and safety representatives, regularly examines and assesses the risks that exist in the daily work routine and provides proper interventions to create a safe work environment. According to the Swedish Work Environment Act employers have main responsibility for the work environment (16). They are required to take all measures needed to prevent the exposure of employees to the risk of ill-health or accidents at work. A central law is the Patient Safety Act that aims to promote high patient safety in healthcare and protect against adverse events (17). Together these laws regulates some aspects of how to promote safe PHM. The value of having a system-oriented approach in healthcare for integrating human factors, safety management and organizational factors, and promoting occupational safety and health, has been emphasized in previous research. One of the most widely used is the systems

For peer review only - http://bmjopen.bmj.com/site/about/guidelines.xhtml

engineering initiative for patient safety, the SEIPS model (18). Understanding and focusing on workplace environment and work processes that have an impact on patient safety, employee safety and health, can help us to target measures correctly. A Swedish guideline for safe PHM has been developed by the PAWSS research group in collaboration with patient organizations, worker unions as well as employer representatives. It will be launched in the end of 2022 by the Swedish Agency for Work Environment Expertise for use in the healthcare sector. The goal of the guideline is to implement evidence-based practice, to promote safe PHM and prevent injuries. The new Swedish guideline will be used in the present study in combination with a strategy for safe PHM at the participating care units. The development and implementation of effective interventions that promote safe PHM are important for employers, HCWs and other stakeholders, since injuries among HCWs and patients are prevalent. Previous research has shown that the risk factors for MSDs among HCWs are multifactorial and interact with one another. Both the physical and mental workload must be taken into consideration when providing individual and organizational interventions (7, 19). The intervention strategies in this study are based on regulations for occupational safety and health, guidelines, and previous research into interventions that promote safe PHM (15, 16, 20, 21). These kinds of interventions are considered as complex interventions. At present, however, there is a lack of knowledge regarding how comprehensive and complex interventions must be tailored to be effective and how learning for safe PHM should be designed to be perceived as useful in healthcare settings. When evaluating complex interventions it is highly important that they are well defined and that both qualitative and quantitative outcomes are used for their evaluation (22).

For peer review only

Aim and hypothesis

The main aim of the cluster randomized controlled trial (RCT) is to implement and evaluate a multifactorial intervention strategy (Arm A) for safe PHM and compare it with a single intervention strategy (Arm B). The trial will be carried out in workplaces in the regional and

municipal healthcare systems. The cluster RCT will evaluate the two strategies with regard to the primary outcome of 1) the **intervention effectiveness** of the applied strategies (safety climate in relation to aspects of PHM) and 2) the **implementation outcome** (perceptions of acceptability, appropriateness and feasibility. The study will evaluate individual health and work aspects. Furthermore, this study aims to: explore **the implementation process** and fidelity to the intervention guidelines; examine the influence of contextual factors (hindrances and facilitators); and explore participants' experiences of working with the multifactorial intervention strategy for safe PHM (Arm A). One tool that will give guidance in this study evaluating the implementation process is the Medical Research Council guidance (MRC guidance) (23). Fidelity is defined as the degree to which the care unit has used the intervention guideline and implemented the intervention strategy as intended by the research team. With a hybrid 2 design, intervention effectiveness and implementation outcome are given equal priority (24). The hypothesis in this study is that care units that receive the multifactorial intervention strategy develop a stronger and more appropriate safety climate with specific focus on PHM compared with units that receive a single intervention strategy. The hypothesis is tested by performing a cluster RCT with pre- and post-measurements among care units in regions and municipalities in Sweden.

METHODS AND ANALYSIS

The study design is a cluster randomized controlled hybrid type 2 trial in which participating care units are randomized into two intervention arms: Arm A or Arm B. The study will be carried out in at least twelve healthcare units in regional and municipal healthcare in Sweden. In regional healthcare, all units treat patients with at least one night stay. Municipal healthcare comprises long term care units for the elderly care. The units will be situated throughout Sweden and thus give a good geographical spread. All recruited care units will be randomized

For peer review only - http://bmjopen.bmj.com/site/about/guidelines.xhtml

at the same time by a computer-generated randomization-list to either Arm A or Arm B after stratification in blocks by the size of the unit (large or small) and type of organization (regional or municipal healthcare). The randomization will be performed by an independent researcher prior to the baseline measurement in order to be able to adjust the planning and information to the care units belonging to Arm A or Arm B.

Sample selection and recruitment

At least six care units will receive a multifactorial intervention strategy (Arm A) and at least six care units will receive a single intervention strategy (Arm B). All care units starts at the same time. A care unit is defined as one which has one manager who is responsible for all employees (HCWs) at the unit. Where a recruited clinic has several care units, they will be counted as one cluster. In nursing homes, a manager often has responsibility for several departments. In such cases, all these departments can be included as one unit in order to prevent risk of so-called contamination. The recruitment of care units and the randomization into two intervention arms (A and B) are presented in Figure 1.

When the study ends with a 12-month measurement, the care units that have a received single intervention strategy will be given access to training concepts for safe PHM and methods for risk assessment. To recruit care units, information will be distributed to workplaces in the Swedish regional and municipal healthcare systems, via contact persons such as managers and Human Resource managers. The research project has the support of a reference group that has been put together for the project and consists of various stakeholders. Information will also be disseminated via advertisements in trade union newspapers and we will highlight the project through newspaper interviews. Several communication channels will be used to recruit units, such as social media and newsletters.

For peer review only - http://bmjopen.bmj.com/site/about/guidelines.xhtml

Patient and public involvement

Key stakeholders, both patient organizations and unions as well as employer representatives and the Swedish work environment authority were consulted to receive input on the guideline for safe PHM that will be implemented in this study. The trial findings will be communicated to all participating healthcare units.

Inclusion Criteria

- Regional healthcare: inpatient units/wards.
- Municipal healthcare: nursing homes for the elderly.

Exclusion Criteria

Care units providing:

- Outpatient home nursing
- Pediatric care
- Parts of Emergency care
- Psychiatric care

Insert figure 1

Recruitment of participants

Prior to start-up, staff from the research group will give further information about the study and the procedure for collecting consent to those care units that have expressed an interest in participating. The HCWs will be invited to participate in the study via the care unit's common email groups or equivalent. They will also be given further information at workplace meetings or equivalent. As a first step the manager will sign a written consent before the care unit's participation in the study. Each care unit that has agreed to participate in the study will have a start-up meeting to introduce the strategy that the care unit has been randomized to and will

For peer review only

work with, Arm A or Arm B. Each HCWs at participating care units will read and sign an informed written consent prior to answering any questionnaires. The cohort will be open, allowing new employees to enter the study. To explore the participants' experiences of the multifactorial intervention strategy for safe PHM, a sub-sample of HCWs in care units who have participated in Arm A will be invited to participate in focus group discussions or individual interviews. Information about the qualitative study will be distributed at the end of the implementation period by means of workplace meetings, emails and the project's website. Prior to participating in individual interviews and focus group discussions, participants will sign a informed consent form.

Intervention strategies for safe PHM

Healthcare units will be randomized to use either a multifactorial intervention strategy (Arm A) or a single intervention strategy (Arm B) for safe PHM. Arm A includes: 1) Swedish guideline for PHM and a digital introduction to using the guideline (two workshops for managers and implementation teams); 2) Training modules (theoretical and practical - one session); 3) Risk assessment with TilThermometer (four times) (12, 25, 26); 4) Fall risk assessment using Downton Fall Risk Index (14) or existing fall risk assessment instrument at the care unit (all patients); and 5) Work environment mapping with Structured Multidisciplinary Work Environment Survey (SMET) (27, 28) including active/personal feedback. Healthcare units randomized to Arm B, will receive the single intervention strategy. They will receive an online introduction to the Swedish guideline for PHM. They will be encouraged to apply the content of the guideline promoting safe PHM in their care unit. No further interventions other than access to the Swedish guideline for PHM and the results from the SMET questionnaire will be presented to managers. Arm A and Arm B will be assigned an external facilitator who provides information, is present at the start-up meeting, and will be the contact person during the study period. All units in Arm B will be contacted by the research team during the 4-month

For peer review only - http://bmjopen.bmj.com/site/about/guidelines.xhtml

implementation period. They will collect contextual data and be able to answer questions concerning the study, but no further implementation support will be given to the care units in Arm B. Both Arm A and Arm B will be encouraged to continue to work with the strategies for safe PHM, integrating them into the ordinary work at the unit until the 12-month follow up.

Implementation strategy

An implementation team will be created at each intervention unit in Arm A. The implementation team will consist of 5-6 people, including the manager, a range of healthcare professions and safety representatives. The care units in Arm A will be offered support from the the external facilitators during the 4-month implementation period to create good conditions for implementation (facilitator/project coordinator). The results from the SMET questionnaire will be presented as feedback to the implementation team along with a dialogue with the manager and discussions about the systematic work environment management. From the 4 months follow up, the results from the SMET questionnaire will be presented to the implementation team again. Training in PHM will be offered to all HCWs. One session of practical training for safe PHM and theoretical education will be offered online. Participating care units implementation team and interested HCWs in Arm A will be able to discuss the Swedish guideline for PHM at two workshops. Here, participants will discuss themes and the guidelines' recommendations. The implementation team will formulate a number of problem areas they want to work with in the workplace using a participatory approach. The TilThermometer will be used for risk assessment four times during the implementation period. Fall risk assessment will be performed on all patients either with Downton Fall Index or existing fall risk assessment instrument at the care unit. Arm A will be encouraged to involve their occupational healthcare services and have an active collaboration during the 4-month implementation period.

For peer review only - http://bmjopen.bmj.com/site/about/guidelines.xhtml

Outcome measures

A mix of quantitative and qualitative methods will be used. The data collection will start 2023 and will be based on questionnaires, interviews and field notes of contextual factors presented in Table 1. Questionnaires will be sent out to HCWs at participating care units and answered digitally at baseline, at 4-month and 12-month follow-up. Background data will be included in the baseline questionnaire, such as age, gender, education, working fulltime/part time, overtime, profession, years of experience in the profession, and current workplace.

Primary outcome measures; The intervention effectiveness will be measured with regard to the safety climate using NOSACQ-50. This comprises 50 items across seven safety climate dimensions in the workplace (management safety priority, commitment, and competence; management safety empowerment; management safety justice; workers' safety commitment; workers' safety priority and risk non-acceptance; safety communication, learning, and trust in co-workers' safety competence; trust in the efficacy of safety systems) (29). The questionnaires use a score with the scale 1-4, where 1 is "Not correct at all" and 4 is "Completely correct". A score of more than 3.30 indicates a good safety climate. An additional 14 specific research-based questions to evaluate the safety climate for PHM will be used in line with previous studies (7). The questions involve dimensions of using equipment, other aspects of safety, performing risk assessment and co-production between caregivers and patients. It uses the scale 1-4, where 1 is "Not correct at all" and 4 is "Completely correct", based on the design in NOSACQ-50 (29). The implementation outcome (acceptability, appropriateness and feasibility) will be measured by a questionnaire to evaluate the usefulness/benefit of the intervention strategies. These are validated instruments with the purpose of assessing the fit and match of a practice or intervention to a given context, targeting different criteria (30). The three measures comprise four items each, answered on 5-

For peer review only - http://bmjopen.bmj.com/site/about/guidelines.xhtml

point Likert-type scales. They have been translated into Swedish and cross-culturally adapted (31). A higher score indicates a better outcome on all three parameters.

Secondary outcome measures; Data collected from questionnaires will include the following secondary outcomes: The "Structured Multidisciplinary Work Environment Survey" (SMET) (27, 28) asks about physically, environmentally and psychosocially demanding work items (30 items). Another question concerns general self-reported health (32). Musculoskeletal pain and symptoms will be measured with questions about the presence of pain and symptoms in the neck, shoulders, elbows, wrists/hands, upper back, lower back, and lower extremities (33). Borg-CR 10 in general, specific for patient handling and movement, and for muscle strength (34, 35) will be used to measure perceived work strain. Individual physical strength is evaluated by the question: How do you evaluate your muscle strength in comparison to others? Data on work ability and work performance will be collected by the Work Ability Index single item, physical and mental demands in work, sick leave, sickness work capacity (36, 37). Furthermore, employees will be asked whether they have experienced any work environment problems affecting health and work performance and sickness presence in the previous seven days (38, 39). Other questions concern the impact of sickness on the ability to work and sickness absence during the previous 12 months: Is your disorder or injury affecting your ability to work? Answers range from no problem to being absent due to sickness. Self-reported sickness absence has in previous studies been shown to have acceptable reliability (40). Four questions are used to evaluate level of physical activity (41, 42).

Implementation outcome

To measure fidelity (to what extent the intervention in Arm A has been followed as intended), as a secondary outcome, a study specific logbook will be used. The logbook contains the five

For peer review only

steps in the intervention strategy: 1) Number of workshops carried out by the manager and implementation team, 2) Number of HCWs attending training modules, 3) Number of risk assessments with TilThermometer, 4) Percentage of fall risk assessments of all patients during the implementation period, 5) Performed SMET with active feedback through study completion. The usefulness of participating in workshops and digital and practical education will be evaluated using a questionnaire.

Implementation process evaluation

Information about the perceived usefulness of the use of strategies for safe PHM and about barriers and facilitators that may influence the implementation process will be collected by inviting managers, implementation teams and HCWs at the care units for focus group discussions with employers and HCWs belonging to Arm A. These will be held after the 4-month study period and before the 12-month follow-up. An interview guide will be developed for each target group of HCWs based on the consolidated framework for implementation research (CFIR) constructs (43).

Table 1. Presentation of outcome measures, method of data collection, data source and time points; T0= baseline, T1= 4 months follow up and T2 12 months follow up.

Measure	Method of data collection	Data source	Time point
Primary outcome			
1. Intervention effectiveness			
Safety climate	Questionnaire (NOSACQ-50) (29)	HCWs	T0, T1 and T2
Safety for patient handling and movement	Questionnaire (research specific)	HCWs	T0, T1 and T2
2. Implementation outcome			
Acceptability	Questionnaire (AIM) (31, 46)	HCWs	T1 and T2
Appropriateness	Questionnaire (IAM) (31, 46)	HCWs	T1 and T2
Feasibility	Questionnaire (FIM) (31, 46)	HCWs	T1 and T2
**Secondary outcome**			
1. Intervention effectiveness			
Evaluation of the work environment	Questionnaire (SMET)(27, 28)	HCWs	T0, T1 and T2
Global Health	Questionnaire (32)	HCWs	T0, T1 and T2
Work Strain	Questionnaire (Borg-CR 10) (34, 35)	HCWs	T0, T1 and T2

For peer review only - http://bmjopen.bmj.com/site/about/guidelines.xhtml

Work performance - work ability	Questionnaire (WAI) (36, 37)	HCWs	T0, T1 and T2
Perceived work environment<br>Production loss<br>Sickness presence<br>Sickness influence on ability to work	Questionnaire (38, 39)	HCWs	T0, T1 and T2
Sickness absence	Questionnaire (40)	HCWs	T0, T1 and T2
Work related musculoskeletal disorders	Questionnaire (33)	HCWs	T0, T1 and T2
Fit for work - physical strength	Questionnaire (single item)	HCWs	T0, T1 and T2
**Physical Activity**<br>Daily physical activity<br>Physical exercise the last 12 months<br>Strenuous physical activity<br>Time of daily physical activity	Questionnaire (41, 42)	HCWs	T0, T1 and T2
Implementation outcome			
Fidelity	Observation, meeting notes, work documents	Process team, research team	During implementation period of 4 months
Experiences of the intervention strategy and implementation process	Meeting notes, logbooks	Managers and implementation team members, HCWs	Between T1 and T2
Experiences of the intervention strategy and implementation process (barriers and facilitators)	Interviews: focus group and individual. CFIR will guide the analysis (45).	Managers and implementation team members, HCWs	Between T1 and T2

*HCWs=healthcare workers (includes workers and managers)

Contextual factors

Contextual factors will be collected from each participating care unit at baseline. These will include: number of risk assessments the previous year; number of managers andHCWs; number of admissions care recipients/places; care burden; number of inpatients; completed work environment training previous year; current work environment policy; completed training in PHM. During the 4-month implementation period a number of explanatory and contextual factors will be collected to be able to explore the implementation process and fidelity to the intervention guidelines. Such factors could, for example, be organizational changes, understaffing, new managers, and crises. These factors will be collected by the

For peer review only

For peer review only - http://bmjopen.bmj.com/site/about/guidelines.xhtml

research team to be able to understand the implementation process and the outcomes of the implemented strategies in all units. They will be collected from both units, Arm A and Arm B, by the research group using field notes, and conversations with managers.

Data analysis and statistics

A power calculation was made to estimate the number of clusters and participants in each cluster. A power level of 0.8 (80%) and alpha 0.05 was used. The calculation resulted in at least six care units (clusters) for each arm with a minimum of 15 participants in each care unit. Two predetermined conditions were taken into consideration in the randomization, size of cluster and regional or municipal healthcare, to ensure equal randomization in Arm A and Arm B.

Quantitative data will be analysed using the IBM Statistical software Package for the Social Sciences (SPSS). Descriptive data are presented with mean and standard deviation, median and range, or counts and percentages. Comparisons between groups are analysed with independent sample t-test, Mann-Whitney U test or chi-2 test. Multiple imputation will be used for missing data. A sensitivity analysis will be made. Potential differences between the intervention arms regarding primary outcome at 4-month and 12-month follow-up will be estimated using linear mixed models for repeated measures. The models will be adjusted for possible confounders (baseline differences concerning age and working experience). The results are presented as point estimates with 95% confidence intervals (CI). Care units with a low rate of adherence to the assigned intervention strategy will in subgroup analysis be compared with units with high rate of adherence. The limit for statistical significance will be set at $\alpha = 0.05$.

All interviews and focus group discussions will be digitally recorded and transcribed verbatim. The transcribed texts will be imported into NVivo 12 (QSRInternational,

For peer review only

For peer review only - http://bmjopen.bmj.com/site/about/guidelines.xhtml

Melbourne, Australia) to manage and code data. Qualitative content analysis will be used to analyse the data (44). A deductive approach will be used and the implementation framework CFIR will guide the analysis in the first step (45). Thereafter, an n inductive analysis will be used to describe the HCWs and the implementation team members' and managers' experiences of the multifaceted intervention strategy for safe PHM with regard to the CFIR constructs more in dept. Data from (log books) activity logs will be compiled and processed qualitatively and quantitatively.

ETHICS AND DISSEMINATION

Ethics approval was received from the national ethical board (Dnr 2021–00578). HCWs working in participating care units will provide informed consent to participate in the study. It will not be possible to identify any individual study participants when the results are presented. All data in paper form, such as questionnaire responses, informed consents, code keys and transcribed interviews, will be handled by the research group with confidentiality and stored in a safe space at the research group organization. A code list will be drawn up and stored separately from the data in a locked cabinet to which only people in the research group who are employed by Region Östergötland have access. Results and characteristics of participating individuals will be presented at group level or in such a way that individuals cannot be identified. A data management plan will be established. All material will be archived in accordance with current legislation and local procedures.

Authors´ contributions

CW is the head of the project and the cluster randomized controlled trial presented in this study protocol. CW, SB, ENS and JF will participate in the execution and data collection of the project. CW, SB, ENS and JS are responsible for the design of the intervention strategy. All co-authors have been involved in the study design. CW and SB wrote the first draft of the

For peer review only - http://bmjopen.bmj.com/site/about/guidelines.xhtml

manuscript and the other co-authors (ENS, JS, JF, MA, PE) were involved in reviewing the manuscript. All authors have read and approved the final manuscript.

DECLARATIONS

Funding: This research was funded by AFA Insurance, Sweden, grant number 190144.

Competing interests: The authors declare that they have no competing interests.

Acknowledgements: We would like to thank the statistician at Linköping University, Mats Fredrikson, for support with the sample size calculation and selection of appropriate statistical analysis.

List of abbreviations

MSD Musculoskeletal disorders

PHM patient handling and movement

HCWs healthcare workers

For peer review only

References

1. Wåhlin C, Kvarnström S, Öhrn A, Nilsing Strid E. Patient and healthcare worker safety risks and injuries. Learning from incident reporting. European Journal of Physiotherapy. 2020;22(1):44-50.

2. Craib K, Hackett G, Back C, Cvitkovich Y, Yassi A. Injury rates, predictors of workplace injuries, and results of an intervention program among community health workers. Public health nursing (boston, mass) [Internet]. 2007; 24(2):[121-31 pp.].

3. Rodriguez-Acosta RL, Richardson DB, Lipscomb HJ, Chen JC, Dement JM, Myers DJ, et al. Occupational injuries among aides and nurses in acute care. American journal of industrial medicine. 2009;52(12):953-64.

4. Keyaerts S, Godderis L, Delvaux E, Daenen L. The association between work-related physical and psychosocial factors and musculoskeletal disorders in healthcare workers: Moderating role of fear of movement. Journal of occupational health. 2022;64(1):e12314.

5. Davis KG, Kotowski SE. Prevalence of Musculoskeletal Disorders for Nurses in Hospitals, Long-Term Care Facilities, and Home Health Care: A Comprehensive Review. Human factors. 2015;57(5):754-92.

6. Pompeii LA, Lipscomb HJ, Schoenfisch AL, Dement JM. Musculoskeletal injuries resulting from patient handling tasks among hospital workers. American Journal of Industrial Medicine. 2009;52(7):571-8.

7. Andersen LL, Vinstrup J, Villadsen E, Jay K, Jakobsen MD. Physical and Psychosocial Work Environmental Risk Factors for Back Injury among Healthcare Workers: Prospective Cohort Study. International journal of environmental research and public health. 2019;16(22).

8. Waters TR, Nelson A, Proctor C. Patient handling tasks with high risk for musculoskeletal disorders in critical care. Critical care nursing clinics of North America. 2007;19(2):131-43.

9. Kugler HL, Taylor NF, Boyd L, Brusco NK. Nurses sustain manual handling risk assessment behaviours six-months after a training program to move patients safely: a pre-post study. Disability and rehabilitation. 2022:1-9.

For peer review only

For peer review only - http://bmjopen.bmj.com/site/about/guidelines.xhtml

10. Eklöf M, Törner M, Pousette A. Organizational and social-psychological conditions in healthcare and their importance for patient and staff safety. A critical incident study among doctors and nurses. Safety Science. 2014;70:211-21.

11. Nilsing Strid E, Wåhlin C, Ros A, Kvarnström S. Health care workers' experiences of workplace incidents that posed a risk of patient and worker injury: a critical incident technique analysis. BMC Health Services Research. 2021;21(1):511.

12. Buck S, Sandqvist J, Nilsing Strid E, Knibbe HJJ, Enthoven P, Wahlin C. Translation and cross-cultural adaptation of the risk assessment instrument TilThermometer for a Swedish version - patient handling in the healthcare sector. BMC musculoskeletal disorders. 2022;23(1):531.

13. LOCOmotion. TilThermometer VVT 2021 [2021-06-16]. Available from: https://www.tilthermometer.com/vvt/.

14. Falls in the elderly Falls in the elderly JH Downton Edward Arnold 158pp £16.95 0-340-54848-7 [Formula: see text]. Nursing standard (Royal College of Nursing (Great Britain) : 1987). 1994;8(19):54.

15. Wahlin C, Stigmar K, Nilsing Strid E. A systematic review of work interventions to promote safe patient handling and movement in the healthcare sector. International journal of occupational safety and ergonomics : JOSE. 2021:1-13.

16. Work Environment Act. Arbetsmiljölag (1977:1160). Svensk Författningssamling, 1977, No. 1160. 1977.

17. Government. S. Patientsäkerhetslag SFS 2010:6 59. [Patient Safety Act SFS 2010:659.] In: Affairs. SMoHaS, editor. 2010.

18. Carayon P, Wetterneck TB, Rivera-Rodriguez AJ, Hundt AS, Hoonakker P, Holden R, et al. Human factors systems approach to healthcare quality and patient safety. Applied ergonomics. 2014;45(1):14-25.

19. Bernal D, Campos-Serna J, Tobias A, Vargas-Prada S, Benavides FG, Serra C. Work-related psychosocial risk factors and musculoskeletal disorders in hospital nurses and nursing aides: a systematic review and meta-analysis. International journal of nursing studies. 2015;52(2):635-48.

20. Dennerlein JT, O'Day ET, Mulloy DF, Somerville J, Stoddard AM, Kenwood C, et al. Lifting and exertion injuries decrease after implementation of an integrated hospital-

For peer review only - http://bmjopen.bmj.com/site/about/guidelines.xhtml

wide safe patient handling and mobilisation programme. Occupational and environmental medicine. 2017;74(5):336-43.

21. Hegewald J, Berge W, Heinrich P, Staudte R, Freiberg A, Scharfe J, et al. Do Technical Aids for Patient Handling Prevent Musculoskeletal Complaints in Health Care Workers?-A Systematic Review of Intervention Studies. International journal of environmental research and public health. 2018;15(3).

22. Campbell M, Fitzpatrick R, Haines A, Kinmonth AL, Sandercock P, Spiegelhalter D, et al. Framework for design and evaluation of complex interventions to improve health. BMJ (Clinical research ed). 2000;321(7262):694-6.

23. Moore GF, Audrey S, Barker M, Bond L, Bonell C, Hardeman W, et al. Process evaluation of complex interventions: Medical Research Council guidance. BMJ (Clinical research ed). 2015;350:h1258.

24. Landes SJ, McBain SA, Curran GM. An introduction to effectiveness-implementation hybrid designs. Psychiatry research. 2019;280:112513.

25. Knibbe JJ, Friele RD. The use of logs to assess exposure to manual handling of patients, illustrated in an intervention study in home care nursing. International Journal of Industrial Ergonomics. 1999;24(4):445-54.

26. Knibbe JJ, Knibbe NE, Waaijer E. Assessments of patients with a 5-category or a 3-category practical classification system: validity and practicality. Work. 2012;41 Suppl 1:5655-6.

27. Haraldsson P, Jonker D, Rolander B, Strengbom E, Areskoug-Josefsson K. Structured Multidisciplinary Work Evaluation Tool (SMET): Reliability testing of a multidisciplinary/multifactorial work questionnaire. Work. 2019;62(2):287-97.

28. Haraldsson P, Jonker D, Strengbom E, Areskoug-Josefsson K. Structured Multidisciplinary work Evaluation Tool: Development and validation of a multidisciplinary work questionnaire. Work. 2016;55:883-91.

29. Kines P, Lappalainen J, Mikkelsen KL, Olsen E, Pousette A, Tharaldsen J, et al. Nordic Safety Climate Questionnaire (NOSACQ-50): A new tool for diagnosing occupational safety climate. International Journal of Industrial Ergonomics. 2011;41(6):634-46.

For peer review only

For peer review only - http://bmjopen.bmj.com/site/about/guidelines.xhtml

30. Weiner BJ, Lewis CC, Stanick C, Powell BJ, Dorsey CN, Clary AS, et al. Psychometric assessment of three newly developed implementation outcome measures. Implementation science : IS. 2017;12(1):108.

31. Bernhardsson S, Boman C, Lundqvist S, Arvidsson D, Börjesson M, Larsson MEH, et al. Implementation of physical activity on prescription for children with obesity in paediatric health care (IMPA): protocol for a feasibility and evaluation study using quantitative and qualitative methods. Pilot and Feasibility Studies. 2022;8(1):117.

32. DeSalvo KB, Jones TM, Peabody J, McDonald J, Fihn S, Fan V, et al. Health care expenditure prediction with a single item, self-rated health measure. Medical care. 2009;47(4):440-7.

33. Kuorinka I, Jonsson B, Kilbom A, Vinterberg H, Biering-Sorensen F, Andersson G, et al. Standardised Nordic questionnaires for the analysis of musculoskeletal symptoms. Applied ergonomics. 1987;18(3):233-7.

34. Borg G. Borg's perceived exertion and pain scales. Champaign, IL, US: Human Kinetics; 1998. viii, 104-viii, p.

35. Borg G. Psychophysical scaling with applications in physical work and the perception of exertion. Scandinavian journal of work, environment & health. 1990;16 Suppl 1:55-8.

36. Roelen CA, van Rhenen W, Groothoff JW, van der Klink JJ, Twisk JW, Heymans MW. Work ability as prognostic risk marker of disability pension: single-item work ability score versus multi-item work ability index. Scandinavian journal of work, environment & health. 2014;40(4):428-31.

37. El Fassi M, Bocquet V, Majery N, Lair ML, Couffignal S, Mairiaux P. Work ability assessment in a worker population: comparison and determinants of Work Ability Index and Work Ability score. BMC public health. 2013;13:305.

38. Strömberg C, Aboagye E, Hagberg J, Bergström G, Lohela-Karlsson M. Estimating the Effect and Economic Impact of Absenteeism, Presenteeism, and Work Environment-Related Problems on Reductions in Productivity from a Managerial Perspective. Value in health : the journal of the International Society for Pharmacoeconomics and Outcomes Research. 2017;20(8):1058-64.

For peer review only

39. Lohela-Karlsson M, Nybergh L, Jensen I. Perceived health and work-environment related problems and associated subjective production loss in an academic population. BMC public health. 2018;18(1):257.

40. Voss M, Stark S, Alfredsson L, Vingård E, Josephson M. Comparisons of self-reported and register data on sickness absence among public employees in Sweden. Occupational and environmental medicine. 2008;65(1):61-7.

41. Olsson SJ, Ekblom O, Andersson E, Borjesson M, Kallings LV. Categorical answer modes provide superior validity to open answers when asking for level of physical activity: A cross-sectional study. Scandinavian journal of public health. 2016;44(1):70-6.

42. Kallings L. Validering av Socialstyrelsens screeningfrågor om fysisk aktivitet. In: Socialstyrelsen, editor. https://www.socialstyrelsen.se/globalassets/sharepoint-dokument/dokument-webb/nationellariktlinjer/levnadsvanor-validering-av-indikatorfragor-till-patienter-om-fysisk-aktivitet.pdf. (InSwedish). 2016.

43. Team-Center. CR. Concolidated framework for implementation research. [

44. Elo S, Kyngas H. The qualitative content analysis process. Journal of advanced nursing. 2008;62(1):107-15.

45. Damschroder LJ, Aron DC, Keith RE, Kirsh SR, Alexander JA, Lowery JC. Fostering implementation of health services research findings into practice: a consolidated framework for advancing implementation science. Implementation science : IS. 2009;4:50.

46. Weiner BJ, Lewis CC, Stanick C, Powell BJ, Dorsey CN, Clary AS, et al. Psychometric assessment of three newly developed implementation outcome measures. Implementation Science. 2017;12(1):108.

For peer review only - http://bmjopen.bmj.com/site/about/guidelines.xhtml

Figure 1. Flowchart diagram showing the steps of recruitment, intervention strategies in Arm A versus arm B and time point for measurement.

For peer review only

For peer review only - http://bmjopen.bmj.com/site/about/guidelines.xhtml

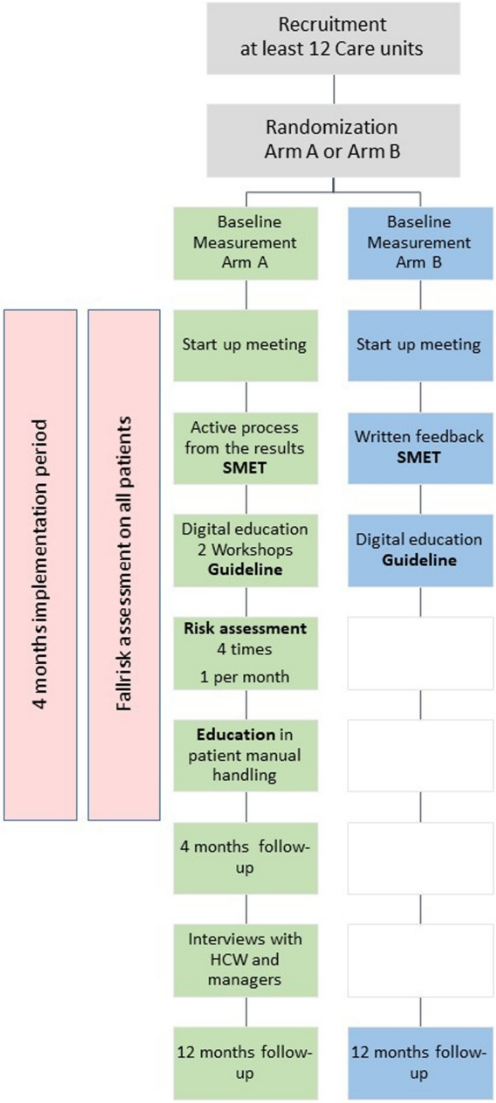

Figure 1. Flowchart diagram showing the steps of recruitment, intervention strategies in Arm A versus arm B and time point for measurement.

190x275mm (300 x 300 DPI)

BMJ Open

Evaluation of the Implementation and Effectiveness of a Multifactorial Intervention Strategy for Safe Patient Handling and Movement in the Healthcare Sector: A Study Protocol of a Cluster Randomised Controlled Trial

Journal:	*BMJ Open*
Manuscript ID	bmjopen-2022-067693.R1
Article Type:	Protocol
Date Submitted by the Author:	10-Jan-2023

Complete List of Authors:	Wahlin, Charlotte; Linköping University, Department of Health, Medicine and Caring Sciences; Karolinska Institutet<br>Buck, Sebastian; Linköping University, Department of Health, Medicine and Caring Sciences<br>Sandqvist, Jan; Linköping University, Department of Health, Medicine and Caring Sciences Rehabilitation and Community Medicine<br>Enthoven, Paul; Linkopings universitet, Department of Health, Medicine and Caring Sciences<br>Fock, Jenni; Linköping University Hospital<br>Andreassen, Maria; Linköping University, Department of Health, Medicine and Caring Sciences Rehabilitation and Community Medicine<br>Strid, Emma; Örebro Universitet

**Primary Subject Heading**:	Occupational and environmental medicine
Secondary Subject Heading:	Evidence based practice
Keywords:	Health & safety < HEALTH SERVICES ADMINISTRATION & MANAGEMENT, PUBLIC HEALTH, Musculoskeletal disorders < ORTHOPAEDIC & TRAUMA SURGERY

SCHOLARONE™
Manuscripts

Evaluation of the Implementation and Effectiveness of a Multifactorial Intervention Strategy for Safe Patient Handling and Movement in the Healthcare Sector: A Study Protocol of a Cluster Randomised Controlled Trial

Charlotte Wåhlin[1, 2], Sebastian Buck[1], Jan Sandqvist[3], Paul Enthoven[4], Jenni Fock[5], Maria Andreassen[3], Emma Nilsing Strid[6]

[1]Occupational and Environmental Medicine Center, Department of Health, Medicine and Caring Sciences, Division of Prevention, Rehabilitation and Community Medicine, Unit of Clinical Medicine, Linköping University, Linköping, Sweden.

[2]Unit of Intervention and Implementation Research for Worker Health, Karolinska Institutet, Stockholm, Sweden.

[3] Department of Health, Medicine and Caring Sciences, Division of Prevention, Rehabilitation and Community Medicine, Unit of Occupational Therapy, Linköping University, Norrköping, Sweden.

[4] Department of Health, Medicine and Caring Sciences, Division of Prevention, Rehabilitation and Community Medicine, Unit of Physiotherapy, Linköping University, Linköping, Sweden.

[5]Linköping University hospital, Region Östergötland, Linköping, Sweden.

[6]University Health Care Research Center, Faculty of Medicine and Health, Örebro University, Örebro, Sweden.

Correspondence to Charlotte Wåhlin: Occupational and Environmental Medicine Center, Linköping University, 58183 Linköping, Sweden.

E-mail: charlotte.wahlin@liu.se

Trial registration ClinicalTrials.gov identifier: NCT05276180

For peer review only

For peer review only - http://bmjopen.bmj.com/site/about/guidelines.xhtml

Abstract

Introduction

Healthcare workers (HCWs) with physically demanding work tasks, such as patient handling and movement (PHM), are at high risk of musculoskeletal disorders. To facilitate safe PHM and prevent musculoskeletal disorders, a combination of workplace interventions, including risk assessments, are needed. The aim of this study is to implement and evaluate a multifactorial intervention strategy for safe PHM and compare it with a single intervention strategy.

Methods and analysis

This cluster randomised controlled trial (RCT) will compare a multifactorial intervention strategy with a single intervention strategy for safe PHM in workplaces in the Swedish regional and municipal healthcare systems. At least twelve healthcare units will be recruited. Care units belonging to Arm A will receive: 1) guidelines for PHM; 2) training modules; 3) risk assessment with TilThermometer; 4) risk assessment with Downtown Fall Risk Index; and 5) work environment mapping. Care units belonging to Arm B will receive interventions 1) and 5). The two strategies will be evaluated with regard to 1) the primary outcome of the applied strategies' intervention effectiveness (safety climate in relation to aspects of PHM) and 2) the primary implementation outcome (acceptability, appropriateness and feasibility). This study will also explore the implementation process and intervention fidelity, examine the influence of contextual factors, and investigate participants' experiences of working with strategies for safe PHM. A mix of quantitative and qualitative methods will be used. The data collection is based on questionnaires, interviews and field notes of contextual factors.

For peer review only - http://bmjopen.bmj.com/site/about/guidelines.xhtml

Ethics and dissemination

The study is approved by the Swedish national ethical board (Dnr 2021–00578). Study results will be published in peer-reviewed journals, presented at conferences and distributed on social media. A lay summary and dissemination strategy will be co-designed with a reference group and participating healthcare units.

Trial registration Number

Trial registration ClinicalTrials.gov identifier: NCT05276180

Keywords: Occupational health, implementation science, risk assessment, moving and lifting patients, health care sector, health care workers, musculoskeletal disorders, occupational safety and health, work environment

Strengths and limitations of this study

- The main strengths of this multicenter project are the randomised controlled trial design with an assessment of both intervention effectiveness and implementation outcomes, and the use of quantitative as well as qualitative methods.
- The findings from this randomised controlled trial will contribute with knowledge about the effectiveness and experience of using two different strategies for promoting safe patient handling and movement at workplaces in healthcare settings.
- Validated outcome measures are used to evaluate intervention effectiveness and implementation outcomes.

For peer review only

77 • The trial findings are limited to workplaces in regional and municipal healthcare and do not include homecare.

For peer review only

For peer review only - http://bmjopen.bmj.com/site/about/guidelines.xhtml

INTRODUCTION

Safe healthcare environment

A safe healthcare environment is vital for both healthcare workers' (HCWs) and patients' health and safety. However, previous research indicates insufficient safety for both HCWs and patients with several risk factors that can potentially cause incidents and injuries (1). Common occupational injuries among HCWs are those caused by sharp objects or needles; exposure to infection; injuries that occur in threatening and violent situations; falls; and injuries that occur during patient handling and movement (PHM) (1-3). Musculoskeletal disorders that occur in connection with PHM are one of the most common injuries, especially in nursing staff. They are most frequent in the lumbar spine, neck and shoulders among nursing staff, especially nurses and assistant nurses (4-7). Patient falls are still the most frequently reported incident; however, these are reducible with multifactorial interventions and education (8, 9). Kugler at al. (10) suggested that patient falls could be prevented with knowledge and skills in assessments of risks for patient and HCW, thereby improving safety in PHM. In addition to strategies for the active management of risks and avoiding injuries, the provision of organisational support is imperative for coping with the consequences when a patient or HCW has been injured (11). Previous research into the relationships between HCW's working conditions and patient injuries demonstrates that a good safety climate supports safe patient care and ensures HCW safety (12), thereby creating a safe healthcare environment. The majority of incidents and injuries among HCWs occur when caring for patients (1). This implies a need to further focus on combining risk assessments for patients and HCWs to prevent and eliminate risks and promote a healthy, safe and sustainable healthcare environment. The importance of performing risk assessments as a basis for choosing interventions is supported by laws and regulations as well as previous research (13, 14).

Risk assessment for safe patient handling and movement

For peer review only

Musculoskeletal disorders resulting from PHM are suggested as preventable by risk assessment methods that identify risk factors or evaluate work techniques (15). One of the more comprehensive methods is TilThermometer, which focuses on the evaluation of physical exposure. The mobility level of patients and use of work equipment are correlated to the physical load on HCWs and therefore the level of exposure (16, 17). The TilThermometer is translated and culturally adapted from Dutch into Swedish and is available online (18, 19). In this study, we will use and evaluate the Swedish version of TilThermometer in healthcare settings. In addition, a safe PHM can also include an assessment of the patient's functioning and risk of falling.

Interventions promoting safe PHM

Musculoskeletal disorders are not only caused by physical exposures; risk factors also include mental workload and organisational aspects, which are multifactorial and interact with each other (6, 20). This complexity must be considered when providing interventions at the individual and organisational levels. In a recent systematic review, interventions including the provision of work equipment and training HCWs were found to increase the HCWs' use of equipment, and peer coaches in safe PHM were associated with fewer occupational injuries. Other promising strategies for safe PHM were participatory ergonomics, joint management and HCW engagement (14). However, further knowledge into how to develop a combination of workplace interventions, including risk assessments, is needed to further facilitate safe PHM and prevent musculoskeletal disorders. The intervention strategies that will be evaluated in this study are based on regulations for occupational safety and health, clinical guidelines, and previous research into interventions that promote safe PHM (13, 14, 21, 22). These intervention strategies can be considered as complex interventions (23), since they include several components, target both HCWs and managers, require their skills and knowledge in PHM, and

For peer review only - http://bmjopen.bmj.com/site/about/guidelines.xhtml

may demand a change in routine practice. A Swedish guideline for safe PHM was launched in December 2022 by the Swedish Agency for Work Environment Expertise for use in the healthcare sector. The goal of the guideline is to implement evidence-based practice, promote safe PHM and prevent injuries. The new Swedish guideline will be used in the present study in combination with a strategy for safe PHM at the participating care units. Further investigation into comprehensive interventions for safe PHM, the implementation process and effectiveness are warranted if we hope to improve the safety for HCWs and patients in PHM.

AIMS AND HYPOTHESIS

The overall aim of the proposed study is to evaluate the implementation and the effectiveness of a multifactorial intervention strategy for safe PHM (A) and compare it with a single intervention strategy (B). The specific aims are as follows: (1) to evaluate and compare the intervention effectiveness of the applied strategies regarding safety climate in relation to PHM; (2) to evaluate the implementation outcome concerning the HCWs' perceptions of acceptability, appropriateness and feasibility of the intervention; and (3) to explore the implementation process and fidelity to the interventions. The implementation process evaluation includes the HCWs' experiences of the multifactorial intervention strategy for safe PHM. The hypothesis in this study is that care units receiving the multifactorial intervention strategy will develop a better safety climate in relation to PHM compared with care units receiving the single intervention strategy. Furthermore, the hypothesis is that the multifactorial intervention strategy will be perceived as more acceptable, appropriate and feasible than the single intervention strategy.

METHODS AND ANALYSIS

Design

For peer review only - http://bmjopen.bmj.com/site/about/guidelines.xhtml

We propose a prospective cluster randomised controlled trial evaluating the implementation and effectiveness of intervention strategies for safe PHM. The study is based on a hybrid 2 trial design, where clinical effectiveness and implementation outcome are given equal priority (24). The implementation process will be evaluated using a combination of qualitative and quantitative methods, as described in the Medical Research Council guidelines for process evaluations of complex interventions (25, 26). For reporting this RCT the study follows the Standard Protocol Items: Recommendations for interventional trials (SPIRIT) checklist (27). The study have taken guidance from the Standards for Reporting Implementation Studies (STaRI).

Setting and participants

The study will be carried out in at least twelve healthcare units in regional and municipal healthcare centres in Sweden. At least six care units will receive the multifactorial intervention strategy (A) and at least six care units will receive the single intervention strategy (B). A care unit is defined as a unit with one manager responsible for all employees (HCWs) at the unit. A clinic or a nursing home with several smaller departments or care units will be included as one unit (cluster) to avoid contamination. We strive to recruit care units situated throughout Sweden to reach geographical spread.

Patient and public involvement

The research project is supported by a reference group consisting of various key stakeholders, both patient organisations and unions as well as employer representatives and the Swedish work environment authority. The group was consulted during the study design and recruitment and also gave input on the guidelines for safe PHM that will be used in this study. After the trial, the trial findings will be communicated to all participating healthcare units.

For peer review only - http://bmjopen.bmj.com/site/about/guidelines.xhtml

Recruitment

To recruit care units, information will be distributed during fall 2022 to workplaces in the Swedish regional and municipal healthcare systems via contact persons such as managers and human resource managers as well as other communication channels such as union newspapers, social media and newsletters. The research group will provide further information about the study and procedures to interested care units. Managers will forward information about the study and participation to the HCWs by e-mail and workplace meetings. To avoid the contamination of the different intervention strategies, no specific information about the content in the respective intervention strategies will be provided to interested care units. To explore the implementation process a subsample of HCWs included in Arm A will be invited to participate in focus group discussions or individual interviews. Information about this qualitative study will be distributed at the end of the implementation period by means of workplace meetings, emails and the project's website.

Inclusion Criteria

- Regional healthcare: inpatient care units with a minimum of 15 HCWs employed at the unit
- Municipal healthcare: nursing homes for older adults with a minimum of 15 HCWs employed at the unit

Exclusion Criteria

Care units providing:

- Outpatient home nursing
- Paediatric care
- Parts of Emergency care
- Psychiatric care

For peer review only - http://bmjopen.bmj.com/site/about/guidelines.xhtml

Figure 1. Flowchart diagram

Procedure

As a first step, the manager will sign an informed written consent form before the care unit's participation in the study. All recruited care units willing to participate will be simultaneously randomised to either Arm A or Arm B by a computer-generated randomisation list. Stratification will be made in blocks by the size of the unit (large or small) and type of organisation (regional or municipal healthcare). Furthermore, care units in the same municipality and within the same regional healthcare departments will also be a factor in the stratification. The randomisation will be performed by an independent statistician prior to the baseline measurement in order to be able to adjust the planning and information to the care units belonging to Arm A or Arm B. The recruitment of care units and the randomisation into two intervention strategies (A and B) are presented in Figure 1. The 12-months study period starts February 2023 and is divided in two phases. The first 4-month is classified as the active implementation period. In the following 8 months the care units will continue to apply the intervention strategy.

A start-up meeting will be held by the research group in the beginning of 2023 at the included care units to introduce the strategy that the care unit has been randomised into. The HCWs at the care units will read and sign an informed written consent prior to answering any questionnaires. The cohort will be open for inclusion, allowing newly employed HCWs to enter the study.

Intervention strategies for safe PHM

For peer review only

Care units will be randomised to use either a multifactorial intervention strategy (Arm A) or a single intervention strategy (Arm B) for safe PHM. Care units in both Arm A and Arm B will base their activities on the Swedish guideline for the promotion of safe PHM in their care unit during meetings, discussions and daily work. Furthermore, all care units will be encouraged to continue to work with the strategies for safe PHM, integrating them into the ordinary work at the unit until the 12-month follow up.

Both arm A and arm B will receive a digital introduction on work environment management focusing on PHM. Arm A includes five components: 1) the Swedish guideline for PHM, a digital introduction to using it for all HCWs and in-depth strategies to discuss during two workshops for managers and implementation teams; 2) training modules (theoretical and practical); 3) risk assessment with TilThermometer (four times at each care unit) (17, 19, 28); 4) fall risk assessment using the Downton Fall Risk Index (DFRI) (29) and daily fall risk assessment instrument at the care unit (all patients); and 5) work environment mapping with the Structured Multidisciplinary Work Environment Survey (SMET) (30, 31), including written and oral feedback to the manager. Care units randomised to Arm B will receive the single intervention strategy, which contains: 1) the Swedish guideline for PHM and a digital introduction for all HCWs to using it and 2) the SMET with written feedback to manager.

Implementation strategy

Arm A and Arm B will be assigned an external facilitator who gives active support to apply the intervention strategy to the care unit during the 4-month implementation period. Furthermore, the external facilitators will be in contact with the care units up until the 12-month follow up. The research group will give active support towards both the external facilitator and care units during the whole study period (12-months). An implementation team will be created in all care units for both Arm A and Arm B. The implementation team will consist of 5-6 HCWs, including the manager, a range of healthcare professions and safety representatives. The implementation

For peer review only - http://bmjopen.bmj.com/site/about/guidelines.xhtml

team will be recommended to have regular meetings to work with their assigned intervention strategy.

In Arm A the results from the baseline SMET questionnaire will be presented as feedback, a dialogue with the manager and discussions about the systematic work environment management and conditions for safe PHM. In the same way, the results from the 4-month follow up will be presented to the manager. Education and training in PHM will be offered to all HCWs in Arm A. One session of web-based theoretical education and practical training for safe PHM will be offered to HCWs in Arm A. Moreover, the implementation team and HCWs in those care units included in Arm A will be able to discuss the Swedish guideline for PHM at two workshops moderated by the research group or persons appointed by the research group. Here, participants will discuss themes and the guidelines' recommendations. The implementation team will formulate several problem areas they want to work with in the workplace using a participatory approach. The TilThermometer will be used for risk assessment four times during the intervention period.

Fall risk assessment will be measured four times using DFRI (all patient's). Daily fall risk assessment is encourage at each unit using DFRI or existing method for fall risk assessment. Arm A will be encouraged to involve their occupational healthcare services and have an active collaboration during the 4-month implementation period. The managers in Arm B will receive written feedback of the results from the baseline SMET questionnaire and at the 4-month follow up. No further implementation strategy will be given except the support from the external facilitator. Arm B will base their strategy on the Swedish guideline for safe PHM and regular meetings. When the study ends with a 12-month measurement (February 2024), the care units

For peer review only - http://bmjopen.bmj.com/site/about/guidelines.xhtml

that have a received single intervention strategy will be given access to training concepts for safe PHM and methods for risk assessment.

Outcome measures

A mix of quantitative and qualitative methods will be used as suggested when evaluating complex interventions (25). The data collection will start 2023 and be based on questionnaires, interviews and field notes of contextual factors, presented in Table 1.

Table 1. Presentation of outcome measures, method of data collection, data source and time points; T0= baseline, T1= 4-month follow up, and T2= 12-month follow up.

Measure	Method of data collection	Data source	Time point
Primary outcome			
1. Intervention effectiveness			
Safety climate	Questionnaire (NOSACQ-50)	HCWs and managers	T0, T1 and T2
Safety for patient handling and movement	Questionnaire (research specific)	HCWs and managers	T0, T1 and T2
2. Implementation outcome			
Acceptability	Questionnaire (AIM)	HCWs and managers	T1 and T2
Appropriateness	Questionnaire (IAM)	HCWs and managers	T1 and T2
Feasibility	Questionnaire (FIM)	HCWs and managers	T1 and T2
**Secondary outcome**			
1. Intervention effectiveness			
Evaluation of the work environment	Questionnaire (SMET)	HCWs and managers	T0, T1 and T2
Global Health	Questionnaire	HCWs and managers	T0, T1 and T2
Work Strain	Questionnaire (Borg-CR 10)	HCWs and managers	T0, T1 and T2
Work performance - work ability	Questionnaire (WAI)	HCWs and managers	T0, T1 and T2
Perceived work environment<br>Production loss<br>Sickness presence<br>Sickness influence on ability to work	Questionnaire	HCWs and managers	T0, T1 and T2
Sickness absence	Questionnaire	HCWs and managers	T0, T1 and T2

For peer review only

For peer review only - http://bmjopen.bmj.com/site/about/guidelines.xhtml

Work related musculoskeletal disorders	Questionnaire	HCWs and managers	T0, T1 and T2
Fit for work - physical strength	Questionnaire (single item)	HCWs and managers	T0, T1 and T2
**Physical Activity**<br>Daily physical activity<br>Physical exercise the last 12 months<br>Strenuous physical activity<br>Time of daily physical activity	Questionnaire	HCWs and managers	T0, T1 and T2
2. Implementation outcome			
Fidelity	Observation, meeting notes, work documents, activity logs	Implementation team, external facilitator, research group	During implementation period of 4 months
Experiences of the intervention strategy and implementation process	Meeting notes, activity logs	Managers and implementation team members, external facilitator HCWs	Between T1 and T2
Experiences of the intervention strategy and implementation process (barriers and facilitators)	Interviews: focus group and individual. CFIR will guide the analysis.	Managers and implementation team members, HCWs	Between T1 and T2

*HCWs=healthcare workers

Questionnaires will be sent out to HCWs at participating care units and answered digitally at baseline, 4-month follow up and 12-month follow up. The baseline questionnaire will include background data, such as age, gender, education, working fulltime/part time, amount of overtime, profession, years of experience in the profession, and current workplace.

Intervention effectiveness

Primary outcomes

The intervention effectiveness will be measured with regard to the safety climate using NOSACQ-50. This comprises 50 items across seven safety climate dimensions in the workplace (management safety priority, commitment, and competence; management safety empowerment; management safety justice; workers' safety commitment; workers' safety

For peer review only

For peer review only - http://bmjopen.bmj.com/site/about/guidelines.xhtml

priority and risk non-acceptance; safety communication, learning, and trust in co-workers' safety competence; and trust in the efficacy of safety systems) (32). The questionnaires are scored on a scale of 1-4, where 1 equals "Strongly disagree" and 4 "Strongly agree", where a score greater than 3.30 indicates a good safety climate. An additional 14 specific research-based questions to evaluate the safety climate for PHM will also be used in line with previous studies (6). These questions involve the dimensions of using equipment, other aspects of safety, performing risk assessment and co-production between caregivers and patients. They also use a scale of 1-4, where 1 is "Strongly disagree" and 4 is "Strongly agree", based on the design in NOSACQ-50 (32).

Secondary outcomes

Data collected from questionnaires will include the following secondary outcomes: SMET (30, 31), which asks about physically, environmentally and psychosocially demanding work items (30 items). Another question concerns general self-reported health (33). Musculoskeletal pain and symptoms will be measured with questions about the presence of pain and symptoms in the neck, shoulders, elbows, wrists/hands, upper back, lower back, and lower extremities (34). The Borg-CR 10 scale, specific for patient handling, movement and muscle strength (35, 36), will be used to measure perceived work strain. Individual physical strength is evaluated by the question: How do you evaluate your muscle strength in comparison to others? Data on work ability and work performance will be collected by the Work Ability Index single item as well as physical and mental demands in work, sick leave, and sickness work capacity (37, 38). Furthermore, HCWs will be asked whether they have experienced any work environment problems affecting health and work performance and sickness presence in the previous seven days (39, 40). Other questions concern the impact of sickness on the ability to work and sickness absence during the previous 12 months, for

For peer review only - http://bmjopen.bmj.com/site/about/guidelines.xhtml

example: Is your disorder or injury affecting your ability to work? Answers range from no problems to absent due to sickness. Self-reported sickness absence has in previous studies been shown to have acceptable reliability (41). Four questions are used to evaluate the level of physical activity (42, 43).

Implementation outcome

Primary outcome

To evaluate the implementation outcome concerning the HCWs' and managers' perceptions of acceptability, appropriateness and feasibility of the intervention strategies, a set of three short questionnaires will be used (44, 45). These are validated questionnaires with the purpose of assessing the fit and match of a practice or intervention to a given context, targeting different criteria (45).The questionnaires comprise four items each, answered on 5-point Likert-type scales. They have been translated into Swedish and cross-culturally adapted (44). A higher score indicates a better outcome on all three parameters.

Secondary outcome

Fidelity is defined as the degree to which the care unit has used the intervention guideline and implemented the intervention strategy as intended by the research team (46). To measure fidelity, a study-specific activity log will be used as recommended in previous research by Bunger at al. (47).

The research group will register for both arms those who attend digital introduction lectures presenting occupational safety and health for the healthcare sector and an introduction to the Swedish guideline on PHM, which is part of the intervention strategy for both Arm A and B. Furthermore, the number of performed meetings with the implementation team at each care unit will be registered.

For peer review only - http://bmjopen.bmj.com/site/about/guidelines.xhtml

In addition, the activity log for arm A contains the five steps outlined in the intervention strategy: 1) the number of meetings and workshops carried out by the manager and implementation team, 2) the number of HCWs attending training modules, 3) the number of risk assessments with TilThermometer, 4) the percentage of fall risk assessments of all patients during the implementation period, and 5) the performed SMET with active feedback through study completion. The evaluation of participating in workshops and digital and practical education will be evaluated using a questionnaire focusing on the usefulness of the content, as well as how new knowledge on work environment and safe PHM can be applied in daily practice.

Implementation process evaluation

Information about the perceived usefulness of the use of strategies for safe PHM and barriers and facilitators that may influence the implementation process will be collected by inviting managers, implementation teams and HCWs at the care units to individual interviews or focus group discussions with employers and HCWs belonging to Arm A. These will be held after the 4-month implementation period and before the 12-month follow up. An interview guide will be developed for each target group of HCWs based on the consolidated framework for implementation research (CFIR) constructs (48).

Contextual factors

Contextual factors will be collected from each participating care unit at baseline. These will include: the number of risk assessments the previous year; the number of managers and HCWs; the number of admissions of care recipients/places; the care burden; the number of inpatients; the amount of completed work environment training previous year; the current work environment policy; any completed training in PHM. During the whole study period, a number of explanatory and contextual factors will be collected to be able to explore the

For peer review only - http://bmjopen.bmj.com/site/about/guidelines.xhtml

implementation process. Such factors could, for example, be organisational changes, understaffing, new managers, and crises. These factors will be collected by the research team and the external facilitator in order to understand the implementation process and the outcomes of the implemented strategies in all care units. They will be collected from care units in both Arm A and Arm B, by the research group using field notes and conversations with managers.

Data analysis and statistics

A power calculation was made to estimate the number of clusters and participants in each cluster. The Power calculation was based on the primary intervention effectiveness outcome NOSACQ-50, and a calculated minimum detectable change of 0.7 with a standard deviation of 0.5 and within cluster correlation of 0.7. A power level of 0.8 (80%) and alpha of 0.05 was used. The calculation resulted in at least six care units (clusters) for each arm with a minimum of 15 participants in each care unit. Two predetermined conditions were taken into consideration in the randomisation, size of cluster and regional or municipal healthcare, as this ensured equal randomisation in Arm A and Arm B.

Quantitative data will be analysed using the IBM Statistical Software Package for the Social Sciences (SPSS). Descriptive data are presented with mean and standard deviation, median and range, or counts and percentages. Comparisons between groups are analysed with an independent sample t-test, Mann-Whitney U test or chi-2 test. Multiple imputation will be used for missing data, and a sensitivity analysis will be made. Potential differences between the intervention arms regarding primary outcome at 4-month and 12-month follow up will be estimated using linear mixed models for repeated measures. The models will be adjusted for possible confounders (baseline differences concerning age and working experience), and the results will be presented as point estimates with 95% confidence intervals (CI). Care units with a low rate of adherence to the assigned intervention strategy will in a subgroup analysis

For peer review only - http://bmjopen.bmj.com/site/about/guidelines.xhtml

be compared with care units with high rate of adherence. The limit for statistical significance will be set at $\alpha = 0.05$. All interviews and focus group discussions will be digitally recorded and transcribed verbatim. The transcribed texts will be imported into NVivo 12 (QSRInternational, Melbourne, Australia) to manage and code data. Qualitative content analysis will be used to analyse the data (49). A deductive approach will be used, and the implementation framework CFIR will guide the analysis in the first step (50). Thereafter, an inductive analysis will be used to describe the HCWs and the implementation of team members' and managers' experiences of the multifaceted intervention strategy for safe PHM with regard to the CFIR constructs in greater depth. Data from activity logs will be compiled and processed qualitatively and quantitatively.

ETHICS AND DISSEMINATION

Ethics approval was received from the Swedish national ethical board (Dnr 2021–00578). HCWs working in participating care units will provide informed consent to participate in the study. It will not be possible to identify any individual study participants when the results are presented. All data in paper form, such as questionnaire responses, informed consents, code keys and transcribed interviews, will be handled by the research group with confidentiality and stored in a safe space at the research group organisation. A code list will be drawn up and stored separately from the data in a locked cabinet to which only people in the research group who are employed by Region Östergötland have access. Moreover, the results and characteristics of participating individuals will be presented at the group level or in such a way that individuals cannot be identified. A data management plan will also be established, and all material will be archived in accordance with current legislation and local procedures. A data monitoring committee was not needed as this is not a clinical trial with a sponsor.

AUTHORS´ CONTRIBUTIONS

For peer review only - http://bmjopen.bmj.com/site/about/guidelines.xhtml

CW is the head of the project and the cluster randomised controlled trial presented in this study protocol. CW, SB, ENS and JF will participate in the execution and collection of the project's data. CW, SB, ENS and JS are responsible for the design of the intervention strategy. All co-authors have been involved in the study design. CW, ENS and SB wrote the first draft of the manuscript, and the other co-authors (JS, JF, MA, PE) were involved in reviewing the manuscript. All authors have read and approved the final manuscript.

DATA AVAILABILITY STATEMENT

A data management plan is included in Trial registration ClinicalTrials.gov identifier: NCT05276180

DECLARATIONS

Funding: This research was funded by AFA Insurance, Sweden, grant number 190144. The funders have no role or authority over the study design; collection, management, analysis or interpretation of data; writing or the decision to submit the report for publication.

Competing interests: The authors declare that they have no competing interests.

Acknowledgements: We would like to thank Mats Fredrikson, a statistician at Linköping University, for support with the sample size calculation and selection of appropriate statistical analysis.

List of abbreviations

PHM patient handling and movement

HCWs healthcare workers

For peer review only - http://bmjopen.bmj.com/site/about/guidelines.xhtml

References

1. Wåhlin C, Kvarnström S, Öhrn A, Nilsing Strid E. Patient and healthcare worker safety risks and injuries. Learning from incident reporting. Euro J Physiother. 2020.
2. Craib K, Hackett G, Back C, Cvitkovich Y, Yassi A. Injury rates, predictors of workplace injuries, and results of an intervention program among community health workers. Public Health Nurs. 2007;24(2):121-31.
3. Rodriguez-Acosta RL, Richardson DB, Lipscomb HJ, Chen JC, Dement JM, Myers DJ, et al. Occupational injuries among aides and nurses in acute care. Am J Ind Med. 2009;52(12):953-64.
4. Davis KG, Kotowski SE. Prevalence of Musculoskeletal Disorders for Nurses in Hospitals, Long-Term Care Facilities, and Home Health Care: A Comprehensive Review. Hum Factors. 2015;57(5):754-92.
5. Pompeii LA, Lipscomb HJ, Schoenfisch AL, Dement JM. Musculoskeletal injuries resulting from patient handling tasks among hospital workers. Am J Ind Med. 2009;52(7):571-8.
6. Andersen LL, Vinstrup J, Villadsen E, Jay K, Jakobsen MD. Physical and Psychosocial Work Environmental Risk Factors for Back Injury among Healthcare Workers: Prospective Cohort Study. Int J Environ Res Public Health. 2019;16(22).
7. Waters TR, Nelson A, Proctor C. Patient handling tasks with high risk for musculoskeletal disorders in critical care. Crit Care Nurs Clin North Am. 2007;19(2):131-43.
8. Cameron ID, Dyer SM, Panagoda CE, Murray GR, Hill KD, Cumming RG, et al. Interventions for preventing falls in older people in care facilities and hospitals. Cochrane Database Syst Rev. 2018;9(9):CD005465.
9. Morris ME, Webster K, Jones C, Hill AM, Haines T, McPhail S, et al. Interventions to reduce falls in hospitals: a systematic review and meta-analysis. Age Ageing. 2022;51(5).
10. Kugler HL, Taylor NF, Boyd L, Brusco NK. Nurses sustain manual handling risk assessment behaviours six-months after a training program to move patients safely: a pre-post study. Disabil Rehabil. 2022:1-9.

For peer review only - http://bmjopen.bmj.com/site/about/guidelines.xhtml

11. Nilsing Strid E, Wåhlin C, Ros A, Kvarnström S. Health care workers' experiences of workplace incidents that posed a risk of patient and worker injury: a critical incident technique analysis. BMC Health Serv Res. 2021;21(1):511.

12. Eklöf M, Törner M, Pousette A. Organizational and social-psychological conditions in healthcare and their importance for patient and staff safety. A critical incident study among doctors and nurses. Saf Sci. 2014;70:211-21.

13. Work Environment Act. Arbetsmiljölag (1977:1160). Svensk Författningssamling, 1977, No. 1160. 1977.

14. Wahlin C, Stigmar K, Nilsing Strid E. A systematic review of work interventions to promote safe patient handling and movement in the healthcare sector. Int J Occ Saf Ergon. JOSE. 2021:1-13.

15. Villarroya A, Arezes P, Díaz-Freijo S, Fraga F. Comparison between five risk assessment methods of patient handling. Int J Indust Ergon. 2016;52:100-8.

16. Knibbe JJ, Knibbe NE. An international validation study of the care thermometer: A tool supporting the quality of ergonomic policies in health care. Work. 2012;41(1):5639-41.

17. Knibbe JJ, Friele RD. The use of logs to assess exposure to manual handling of patients, illustrated in an intervention study in home care nursing. Int J Indust Ergon. 1999;24(4):445-54.

18. LOCOmotion. TilThermometer VVT 2021 [2021-06-16]. Available from: https://www.tilthermometer.com/vvt/.

19. Buck S, Sandqvist J, Nilsing Strid E, Knibbe HJJ, Enthoven P, Wahlin C. Translation and cross-cultural adaptation of the risk assessment instrument TilThermometer for a Swedish version - patient handling in the healthcare sector. BMC Musculoskel Disord. 2022;23(1):531.

20. Bernal D, Campos-Serna J, Tobias A, Vargas-Prada S, Benavides FG, Serra C. Work-related psychosocial risk factors and musculoskeletal disorders in hospital nurses and nursing aides: a systematic review and meta-analysis. Int J Nurs Stud. 2015;52(2):635-48.

21. Dennerlein JT, O'Day ET, Mulloy DF, Somerville J, Stoddard AM, Kenwood C, et al. Lifting and exertion injuries decrease after implementation of an integrated hospital-

For peer review only - http://bmjopen.bmj.com/site/about/guidelines.xhtml

wide safe patient handling and mobilisation programme. Occ Environ Med. 2017;74(5):336-43.

22. Hegewald J, Berge W, Heinrich P, Staudte R, Freiberg A, Scharfe J, et al. Do Technical Aids for Patient Handling Prevent Musculoskeletal Complaints in Health Care Workers?-A Systematic Review of Intervention Studies. Int J Environ Res Pub Health. 2018;15(3).

23. Campbell M, Fitzpatrick R, Haines A, Kinmonth AL, Sandercock P, Spiegelhalter D, et al. Framework for design and evaluation of complex interventions to improve health. BMJ (Clin Res Ed). 2000;321(7262):694-6.

24. Curran GM, Bauer M, Mittman B, Pyne JM, Stetler C. Effectiveness-implementation hybrid designs: combining elements of clinical effectiveness and implementation research to enhance public health impact. Med care. 2012;50(3):217-26.

25. Moore GF, Audrey S, Barker M, Bond L, Bonell C, Hardeman W, et al. Process evaluation of complex interventions: Medical Research Council guidance. BMJ : Brit Med J. 2015;350:h1258.

26. Skivington K, Matthews L, Simpson SA, Craig P, Baird J, Blazeby JM, et al. A new framework for developing and evaluating complex interventions: update of Medical Research Council guidance. BMJ (Clin Res Ed). 2021;374:n2061.

27. Chan AW, Tetzlaff JM, Altman DG, Laupacis A, Gøtzsche PC, Krleža-Jerić K, Hróbjartsson A, Mann H, Dickersin K, Berlin JA, Doré CJ, Parulekar WR, Summerskill WS, Groves T, Schulz KF, Sox HC, Rockhold FW, Rennie D, Moher D. SPIRIT 2013 statement: defining standard protocol items for clinical trials. Ann Intern Med. 2013 Feb 5;158(3):200-7.

28. Knibbe JJ, Knibbe NE, Waaijer E. Assessments of patients with a 5-category or a 3-category practical classification system: validity and practicality. Work. 2012;41 Suppl 1:5655-6.

29. Falls in the elderly Falls in the elderly JH Downton Edward Arnold 158pp £16.95 0-340-54848-7 [Formula: see text]. Nursing (Royal College of Nursing (Great Britain) : 1987). 1994;8(19):54.

For peer review only

30. Haraldsson P, Jonker D, Rolander B, Strengbom E, Areskoug-Josefsson K. Structured Multidisciplinary Work Evaluation Tool (SMET): Reliability testing of a multidisciplinary/multifactorial work questionnaire. Work. 2019;62(2):287-97.

31. Haraldsson P, Jonker D, Strengbom E, Areskoug-Josefsson K. Structured Multidisciplinary work Evaluation Tool: Development and validation of a multidisciplinary work questionnaire. Work. 2016;55:883-91.

32. Kines P, Lappalainen J, Mikkelsen KL, Olsen E, Pousette A, Tharaldsen J, et al. Nordic Safety Climate Questionnaire (NOSACQ-50): A new tool for diagnosing occupational safety climate. Int J Indust Ergon. 2011;41(6):634-46.

33. DeSalvo KB, Jones TM, Peabody J, McDonald J, Fihn S, Fan V, et al. Health care expenditure prediction with a single item, self-rated health measure. Med care. 2009;47(4):440-7.

34. Kuorinka I, Jonsson B, Kilbom A, Vinterberg H, Biering-Sorensen F, Andersson G, et al. Standardised Nordic questionnaires for the analysis of musculoskeletal symptoms. Appl Ergon. 1987;18(3):233-7.

35. Borg G. Borg's perceived exertion and pain scales. Champaign, IL, US: J Hum Kinet; 1998. viii, 104-viii, p.

36. Borg G. Psychophysical scaling with applications in physical work and the perception of exertion. Scand J Work Environ Health. 1990;16 Suppl 1:55-8.

37. Roelen CA, van Rhenen W, Groothoff JW, van der Klink JJ, Twisk JW, Heymans MW. Work ability as prognostic risk marker of disability pension: single-item work ability score versus multi-item work ability index. Scand J Work Environ Health. 2014;40(4):428-31.

38. El Fassi M, Bocquet V, Majery N, Lair ML, Couffignal S, Mairiaux P. Work ability assessment in a worker population: comparison and determinants of Work Ability Index and Work Ability score. BMC Pub Health. 2013;13:305.

39. Strömberg C, Aboagye E, Hagberg J, Bergström G, Lohela-Karlsson M. Estimating the Effect and Economic Impact of Absenteeism, Presenteeism, and Work Environment-Related Problems on Reductions in Productivity from a Managerial Perspective. Value in health : J Int Soc for Pharmacoecon Outcome Res. 2017;20(8):1058-64.

For peer review only - http://bmjopen.bmj.com/site/about/guidelines.xhtml

40. Lohela-Karlsson M, Nybergh L, Jensen I. Perceived health and work-environment related problems and associated subjective production loss in an academic population. BMC Pub Health. 2018;18(1):257.

41. Voss M, Stark S, Alfredsson L, Vingård E, Josephson M. Comparisons of self-reported and register data on sickness absence among public employees in Sweden. Occ Environ Med. 2008;65(1):61-7.

42. Olsson SJ, Ekblom O, Andersson E, Borjesson M, Kallings LV. Categorical answer modes provide superior validity to open answers when asking for level of physical activity: A cross-sectional study. Scand J Pub Health. 2016;44(1):70-6.

43. Kallings L. Validering av Socialstyrelsens screeningfrågor om fysisk aktivitet. In: Socialstyrelsen, editor. https://www.socialstyrelsen.se/globalassets/sharepoint-dokument/dokument-webb/nationellariktlinjer/levnadsvanor-validering-av-indikatorfragor-till-patienter-om-fysisk-aktivitet.pdf. (In Swedish). 2016.

44. Bernhardsson S, Boman C, Lundqvist S, Arvidsson D, Börjesson M, Larsson MEH, et al. Implementation of physical activity on prescription for children with obesity in paediatric health care (IMPA): protocol for a feasibility and evaluation study using quantitative and qualitative methods. Pilot Feasibility Stud. 2022;8(1):117.

45. Weiner BJ, Lewis CC, Stanick C, Powell BJ, Dorsey CN, Clary AS, et al. Psychometric assessment of three newly developed implementation outcome measures. Implement Sci. 2017;12(1):108.

46. Landes SJ, McBain SA, Curran GM. An introduction to effectiveness-implementation hybrid designs. Psych Res. 2019;280:112513.

47. Bunger AC, Powell BJ, Robertson HA, MacDowell H, Birken SA, Shea C. Tracking implementation strategies: a description of a practical approach and early findings. Health Res Policy Syst. 2017;15(1):15.

48. Team-Center. CR. Consolidated framework for implementation research.

49. Elo S, Kyngas H. The qualitative content analysis process. J Adv Nurs. 2008;62(1):107-15.

50. Damschroder LJ, Aron DC, Keith RE, Kirsh SR, Alexander JA, Lowery JC. Fostering implementation of health services research findings into practice: a consolidated framework for advancing implementation science. Implement Sci : IS. 2009;4:50.

For peer review only - http://bmjopen.bmj.com/site/about/guidelines.xhtml

Figure 1. Flowchart diagram showing the steps of recruitment, intervention strategies in Arm A versus arm B and time point for measurement.

For peer review only

For peer review only - http://bmjopen.bmj.com/site/about/guidelines.xhtml

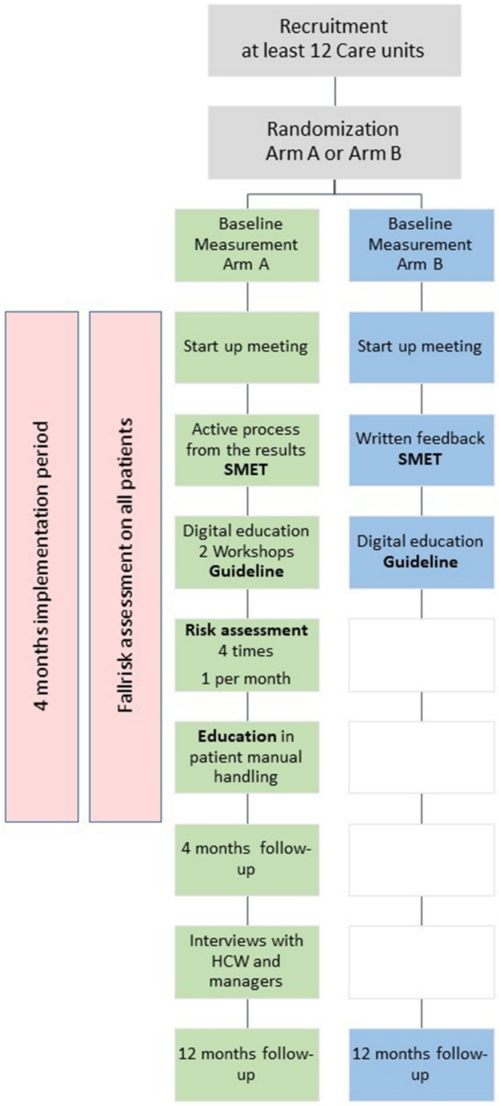

Figure 1. Flowchart diagram showing the steps of recruitment, intervention strategies in Arm A versus arm B and time point for measurement.

190x275mm (300 x 300 DPI)

SPIRIT
STANDARD PROTOCOL ITEMS: RECOMMENDATIONS FOR INTERVENTIONAL TRIALS

SPIRIT 2013 Checklist: Recommended items to address in a clinical trial protocol and related documents*

Section/item	Item No	Description	Addressed on page number
**Administrative information**			
Title	1	Descriptive title identifying the study design, population, interventions, and, if applicable, trial acronym	____1_________
Trial registration	2a	Trial identifier and registry name. If not yet registered, name of intended registry	___1,3________
	2b	All items from the World Health Organization Trial Registration Data Set	____N/A______
Protocol version	3	Date and version identifier	_______1______
Funding	4	Sources and types of financial, material, and other support	_______19______
Roles and responsibilities	5a	Names, affiliations, and roles of protocol contributors	________19_____
	5b	Name and contact information for the trial sponsor	________N/A____
	5c	Role of study sponsor and funders, if any, in study design; collection, management, analysis, and interpretation of data; writing of the report; and the decision to submit the report for publication, including whether they will have ultimate authority over any of these activities	________N/A__
	5d	Composition, roles, and responsibilities of the coordinating centre, steering committee, endpoint adjudication committee, data management team, and other individuals or groups overseeing the trial, if applicable (see Item 21a for data monitoring committee)	________18_____

For peer review only

**Introduction**			
Background and rationale	6a	Description of research question and justification for undertaking the trial, including summary of relevant studies (published and unpublished) examining benefits and harms for each intervention	____4-6________
	6b	Explanation for choice of comparators	_______5______
Objectives	7	Specific objectives or hypotheses	_______6______
Trial design	8	Description of trial design including type of trial (eg, parallel group, crossover, factorial, single group), allocation ratio, and framework (eg, superiority, equivalence, noninferiority, exploratory)	_______7______
**Methods: Participants, interventions, and outcomes**			
Study setting	9	Description of study settings (eg, community clinic, academic hospital) and list of countries where data will be collected. Reference to where list of study sites can be obtained	_____7_______
Eligibility criteria	10	Inclusion and exclusion criteria for participants. If applicable, eligibility criteria for study centres and individuals who will perform the interventions (eg, surgeons, psychotherapists)	_____8_______
Interventions	11a	Interventions for each group with sufficient detail to allow replication, including how and when they will be administered	____9-10_______
	11b	Criteria for discontinuing or modifying allocated interventions for a given trial participant (eg, drug dose change in response to harms, participant request, or improving/worsening disease)	____N/A______
	11c	Strategies to improve adherence to intervention protocols, and any procedures for monitoring adherence (eg, drug tablet return, laboratory tests)	___ 14_______
	11d	Relevant concomitant care and interventions that are permitted or prohibited during the trial	_______N/A______
Outcomes	12	Primary, secondary, and other outcomes, including the specific measurement variable (eg, systolic blood pressure), analysis metric (eg, change from baseline, final value, time to event), method of aggregation (eg, median, proportion), and time point for each outcome. Explanation of the clinical relevance of chosen efficacy and harm outcomes is strongly recommended	_____11-14_
Participant timeline	13	Time schedule of enrolment, interventions (including any run-ins and washouts), assessments, and visits for participants. A schematic diagram is highly recommended (see Figure)	____figure 1____

For peer review only - http://bmjopen.bmj.com/site/about/guidelines.xhtml

Sample size	14	Estimated number of participants needed to achieve study objectives and how it was determined, including clinical and statistical assumptions supporting any sample size calculations	______17_______
Recruitment	15	Strategies for achieving adequate participant enrolment to reach target sample size	_______8______
**Methods: Assignment of interventions (for controlled trials)**			
Allocation:			
Sequence generation	16a	Method of generating the allocation sequence (eg, computer-generated random numbers), and list of any factors for stratification. To reduce predictability of a random sequence, details of any planned restriction (eg, blocking) should be provided in a separate document that is unavailable to those who enrol participants or assign interventions	_______9_____
Allocation concealment mechanism	16b	Mechanism of implementing the allocation sequence (eg, central telephone; sequentially numbered, opaque, sealed envelopes), describing any steps to conceal the sequence until interventions are assigned	_______9______
Implementation	16c	Who will generate the allocation sequence, who will enrol participants, and who will assign participants to interventions	______9_______
Blinding (masking)	17a	Who will be blinded after assignment to interventions (eg, trial participants, care providers, outcome assessors, data analysts), and how	_____No blinding
	17b	If blinded, circumstances under which unblinding is permissible, and procedure for revealing a participant's allocated intervention during the trial	____N/A_______
**Methods: Data collection, management, and analysis**			
Data collection methods	18a	Plans for assessment and collection of outcome, baseline, and other trial data, including any related processes to promote data quality (eg, duplicate measurements, training of assessors) and a description of study instruments (eg, questionnaires, laboratory tests) along with their reliability and validity, if known. Reference to where data collection forms can be found, if not in the protocol	__12-15, table 1
	18b	Plans to promote participant retention and complete follow-up, including list of any outcome data to be collected for participants who discontinue or deviate from intervention protocols	______N/A____

Data management	19	Plans for data entry, coding, security, and storage, including any related processes to promote data quality (eg, double data entry; range checks for data values). Reference to where details of data management procedures can be found, if not in the protocol	_______18____
Statistical methods	20a	Statistical methods for analysing primary and secondary outcomes. Reference to where other details of the statistical analysis plan can be found, if not in the protocol	______17________
	20b	Methods for any additional analyses (eg, subgroup and adjusted analyses)	_______17_______
	20c	Definition of analysis population relating to protocol non-adherence (eg, as randomised analysis), and any statistical methods to handle missing data (eg, multiple imputation)	________17______

Methods: Monitoring

Data monitoring	21a	Composition of data monitoring committee (DMC); summary of its role and reporting structure; statement of whether it is independent from the sponsor and competing interests; and reference to where further details about its charter can be found, if not in the protocol. Alternatively, an explanation of why a DMC is not needed	________18____
	21b	Description of any interim analyses and stopping guidelines, including who will have access to these interim results and make the final decision to terminate the trial	________N/A____
Harms	22	Plans for collecting, assessing, reporting, and managing solicited and spontaneously reported adverse events and other unintended effects of trial interventions or trial conduct	_______N/A_______
Auditing	23	Frequency and procedures for auditing trial conduct, if any, and whether the process will be independent from investigators and the sponsor	________N/A______

Ethics and dissemination

Research ethics approval	24	Plans for seeking research ethics committee/institutional review board (REC/IRB) approval	_______18_______
Protocol amendments	25	Plans for communicating important protocol modifications (eg, changes to eligibility criteria, outcomes, analyses) to relevant parties (eg, investigators, REC/IRBs, trial participants, trial registries, journals, regulators)	_______N/A____

For peer review only - http://bmjopen.bmj.com/site/about/guidelines.xhtml

Consent or assent	26a	Who will obtain informed consent or assent from potential trial participants or authorised surrogates, and how (see Item 32)	______18_______
	26b	Additional consent provisions for collection and use of participant data and biological specimens in ancillary studies, if applicable	______N/A______
Confidentiality	27	How personal information about potential and enrolled participants will be collected, shared, and maintained in order to protect confidentiality before, during, and after the trial	_______18_____
Declaration of interests	28	Financial and other competing interests for principal investigators for the overall trial and each study site	______ 19________
Access to data	29	Statement of who will have access to the final trial dataset, and disclosure of contractual agreements that limit such access for investigators	_______19______
Ancillary and post-trial care	30	Provisions, if any, for ancillary and post-trial care, and for compensation to those who suffer harm from trial participation	_______N/A______
Dissemination policy	31a	Plans for investigators and sponsor to communicate trial results to participants, healthcare professionals, the public, and other relevant groups (eg, via publication, reporting in results databases, or other data sharing arrangements), including any publication restrictions	________3____
	31b	Authorship eligibility guidelines and any intended use of professional writers	______18_______
	31c	Plans, if any, for granting public access to the full protocol, participant-level dataset, and statistical code	______19_______
**Appendices**			
Informed consent materials	32	Model consent form and other related documentation given to participants and authorised surrogates	______N/A______
Biological specimens	33	Plans for collection, laboratory evaluation, and storage of biological specimens for genetic or molecular analysis in the current trial and for future use in ancillary studies, if applicable	_______N/A______

*It is strongly recommended that this checklist be read in conjunction with the SPIRIT 2013 Explanation & Elaboration for important clarification on the items. Amendments to the protocol should be tracked and dated. The SPIRIT checklist is copyrighted by the SPIRIT Group under the Creative Commons "Attribution-NonCommercial-NoDerivs 3.0 Unported" license.
